# Birth Weight and Subsequent Risk of Total Leukemia and Acute Leukemia: A Systematic Review and Meta-Analysis

**DOI:** 10.3389/fped.2021.722471

**Published:** 2021-09-23

**Authors:** Hailuo Che, Dunmei Long, Qian Sun, Lina Wang, Yunbin Li

**Affiliations:** ^1^Department of Obstetrics, Zaozhuang Municipal Hospital, Zaozhuang, China; ^2^Department of Obstetrics and Gynecology, Maternal and Child Health Care of Zaozhuang, Zaozhuang, China; ^3^Department of Obstetrics, Maternal and Child Health Care of Zaozhuang, Zaozhuang, China; ^4^Department of Obstetricsl, Shanting District People's Hospital, Zaozhuang, China; ^5^Department of Hematology, Zaozhuang Municipal Hospital, Zaozhuang, China

**Keywords:** leukemia, acute leukemia, meta-analysis, high birth weight, low birth weight

## Abstract

**Objective:** Birth weight, an important indicator of fetal nutrition and degree of development, may affect the risk of subsequent leukemia. At present, little is known about the effect of birth weight on acute myeloid leukemia (AML) and whether there is a dose-dependent relationship of birth weight with acute lymphoid leukemia (ALL) and AML. To address these questions, the present work aimed to systematically investigate the relationship between birth weight and the risk of subsequent leukemia based on the current epidemiological studies

**Methods:** Relevant studies were systematically retrieved from electronic databases PubMed, Embase, and Cochrane Library, from inception to May 15th, 2021. Finally, 28 studies (including 21 case-control studies and 7 cohort studies) were included for the final meta-analysis. Results in cohort studies were performed by risk ratios (RRs), while those in case-control studies by odds ratios (ORs), and all results were assessed by adopting the random-effect model. Besides, a dose-dependent analysis was conducted based on the cohort studies.

**Results:** Compared with the population with normal birth weight (NBW), the population with high birth weight (HBW) might have an increased risk of leukemia (OR 1.33, 95%CI 1.20–1.49; *I*^2^ 0%). Meanwhile, low birth weight (LBW) was associated with a decreased risk of ALL, as evidenced from the pooled analysis of case-control studies (OR 0.83, 95% CI 0.75–0.92; *I*^2^ 23.3%). However, relative to NBW population, the HBW population might have an increased risk of ALL (OR 1.28, 95% CI 1.20–1.35; *I*^2^ 7%). There was no obvious evidence supporting the relationship between LBW and the risk of AML from the pooled analysis of case-control studies (OR, 1.11 95% CI 0.87–1.42; *I*^2^ 31.7%).

**Conclusions:** Overall, in children and young adults, HBW population may be associated with the risks of subsequent leukemia and AML relative to NBW population, but the supporting dose-dependent evidence is lacking. In addition, compared with NBW population, there is stronger evidence supporting a significantly increased risk of subsequent ALL in HBW population, and a decreased risk in LBW population in a dose-dependent manner. More prospective studies with large samples are warranted in the future to validate and complement these findings.

## Introduction

Leukemia is a malignant clonal disease of hematopoietic stem cells (HSCs). It is characterized by the uncontrolled proliferation and development of leukocytes in the bone marrow and peripheral blood, which in turn invade the internal organs, such as the liver and spleen (1–3). Leukemia, a common hematologic tumor, remains the most common cancer in children. In general, leukemia can be classified into acute and chronic subtypes according to the disease progression degree, of them, acute leukemia is the most common in clinical work. Acute leukemia encompasses acute lymphoid leukemia (ALL) and acute myeloid leukemia (AML). Typically, ALL has been reported to account for approximately 80% of all the diagnosed leukemia cases in children aged 0–19 years, while AML accounts for 15–20% (4). Some perinatal features (such as gestational age, gender, and birth order) and maternal features (like age) are associated with the risk of childhood leukemia. However, the associations of other features with leukemia, especially with the leukemia subtypes, remain to be further elucidated. Currently, the pathogenesis of leukemia is unclear. Typically, exposure early in life may lead to dramatic health consequences, including the risk of cancer in the childhood and throughout the life of an individual (5).

Birth weight, an important indicator of fetal nutrition and degree of development, may affect the risk of subsequent leukemia. Although several previous studies have suggested that high birth weight (HBW) increases the risk of subsequent ALL, the results are inconsistent (6–9). Nevertheless, little is known about the effect of birth weight on AML and whether there is a dose-dependent relationship between birth weight and ALL/AML. To address these questions, this study aimed to systematically investigate the relationship between birth weight and the risk of subsequent leukemia based on existing epidemiological studies.

## Methods

This meta-analysis was conducted according to the guidance of Meta-analyses of Observational Studies in Epidemiology (MOOSE) (10). Two investigators (Che and Long) systematically searched the electronic databases PubMed, Embase, and Cochrane Library, from inception to May 15th, 2021, without language restriction. Two groups of medical subject terms (MeSH), including “birth weight” and “leukemia,” were used for study search. Meanwhile, Boolean operator “OR” was used within groups, whereas “AND” was used between groups. To identify more relevant studies, the library entries were retrieved manually. Besides, previous meta-analyses were also reviewed if applicable. The detailed search flow is displayed in Appendix 1 (Supplementary Material).

### Study Selection

Inclusion criteria were determined following the PICOS standards: (1) The study population did not have any family history of cancers, exposure to radiation and chemicals, or congenital disease (such as Down's syndrome), with birth weight being the interested exposure. (2) Different birth weight levels were compared. (3) One birth weight group served as the control or reference group. (4) The study outcomes reported the incidence of leukemia. (5) The study types were restricted to case-controlled, cohort studies, or randomized controlled trials (RCTs). (6) The related odds ratios (ORs), risk ratios (RRs), or hazard ratios (HRs) and corresponding 95% confidence intervals (CIs) were available or might be calculated. Meanwhile, the exclusion criteria were as follows: (1) The study population had a family history of cancers, exposure to radiation and chemicals, or congenital disease like Down's syndrome, and birth weight was not the interested exposure. (2) The studies did not report the risk of leukemia or acute leukemia by comparing different birth weight levels. (3) Cross-sectional studies, pooled studies, case reports or series, conference abstracts should be excluded. (4) Related ORs, RRs, or HRs were not obtained or converted.

### Data Extraction and Quality Assessment

An unified list was used to extract the following baseline data from the included studies, including first author, publication year, country, sample size, date of birth or diagnosis, BMI categories, types of leukemia, ascertainment of leukemia, and maximum adjusted variables. The maximum variables adjusted for ORs, RRs, or HRs were extracted. Any disagreements were solved by the opinion from a third investigator. The quality of included studies was evaluated by using the Newcastle-Ottawa Scale (NOS) items, with the total score of 9 stars (11). Studies with a score ≥6 stars were considered as high-quality studies, otherwise, they were the low-quality studies.

### Statistical Analysis

Referring to most of the included studies, this study defined birth weight ≤2,500 g, 2,500–4,000 g, ≥4,000 g as “low birth weight (LBW),” “normal birth weight (NBW),” and “HBW,” respectively. When one study reported different LBWs or HBWs (such as ≤ 2,000 g, 2,000–2,500, 4,000–4,500 g, ≥4,500 g), it was analyzed separately. The primary endpoint of our study was to qualitatively analyze the relationship between birth weight and leukemia/acute leukemia (like LBW vs. NBW, HBW vs. NBW). Generally speaking, HRs can be roughly considered to be equal to RRs in cohort studies (12). Therefore, results of all the cohort studies were performed by RRs, whereas those of case-control studies by ORs. Statistical heterogeneity was evaluated using *I*^2^ statistics, with the *I*^2^ values of 25, 50, and 75% indicating low, moderate, and high inconsistency, respectively (13). If there were high heterogeneities between studies, subgroup and sensitivity analyses were performed to explore the possible sources of heterogeneity between groups, and meta-regression analysis was further carried out in the case of enough included studies (*n* > 10). This study used a random-effects model to more conservatively estimate the pooled RRs and ORs, since more robust results were obtained after aggregating with this model. In addition, the risk of potential publication bias was assessed by funnel plots as well as Begg's and Egger's tests (14, 15). Trim and fill analyses were performed if necessary.

The secondary endpoint of this study was to quantitatively assess the effect of birth weight on leukemia. For this purpose, a dose-response meta-analysis was performed on the included cohort studies. To maximally include the available cohort studies, the robust error meta-regression method described by Xu and Doi (16) was utilized to establish a potential dose-response relationship between birth weight and leukemia. In this “one-stage” framework approach, each of the included studies was considered as a cluster across the whole population, so long as the study included at least two categories.

In this paper, the restricted cubic spline was utilized to fit the potential non-linear trend with 3 knots, and non-linear *p*-values were calculated by testing the second spline coefficients to zero. The non-linear model was adopted when the non-linear *p* < 0.05; otherwise, the linear model was used. In general, the lowest-dose category should be used as a reference in the included studies; however, when the non-lowest-dose studies were used as reference, they were converted via an Excel macro file produced by Hamling et al. (17) based on the Greenland and Longnecker's (18) theory. The corresponding authors were contacted when the number of cases in a particular category was missing. Also, when the open intervals were studied, the amplitude was assumed to be the same as the adjacent category or 1.2-fold of the node (19). All statistical analyses were performed by Stata 12.0E.

## Results

**Figure 1:**
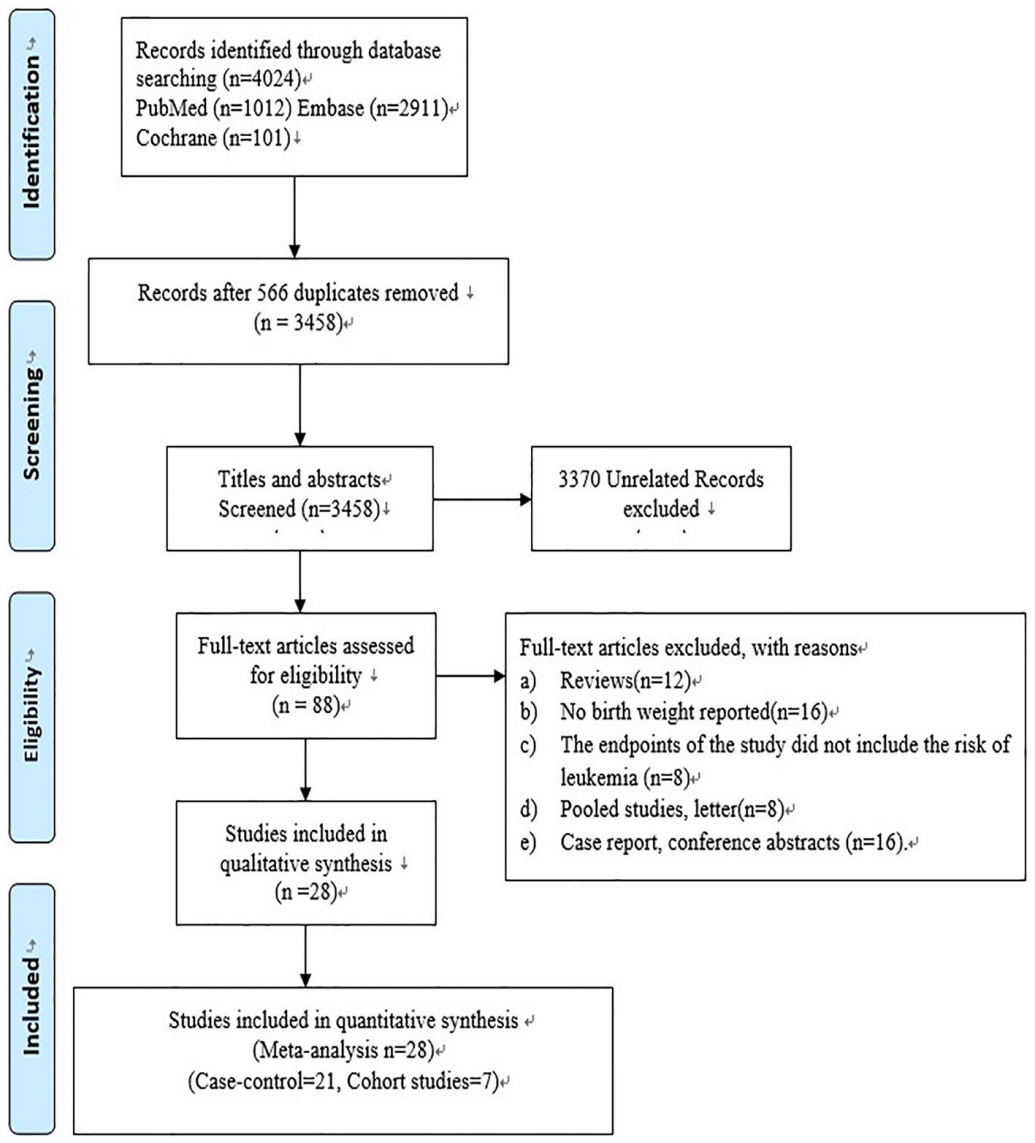
Flow chart of search results.

At first, a total of 4,024 studies were included. After removing 566 duplicated studies, 3,458 studies were retained. Then, the titles and abstracts of these 3,458 studies were read, and 3,370 unrelated studies were further excluded; as a result, only 88 studies were left for full-text review. Finally, only 28 studies (including 21 case-control and 7 cohort studies) were included, as shown in Figure 1. The specific reasons of exclusion were listed as follows: (1) reviews (*n* = 12); (2) no available information of birth weight (*n* = 16); (3) the study endpoints did not include the risk of leukemia (*n* = 8); (4) pooled studies, letters (*n* = 8); and (5) case reports, conference abstracts (*n* = 16).

In the 28 studies, participants were children and young adults <29 years of age. Among the 21 case-control studies (6–9, 20–36), 21 involving altogether 111,643 participants (both cases and controls) reported the association between birth weight and the risk of ALL; 14 including 82,566 participants (both cases and controls) mentioned the relationship between birth weight and the risk of AML; while 7 containing 43,501 participants (both cases and controls) reported the association between birth weight and the risk of total leukemia. Among the 7 cohort studies (37–43), 5 recruiting altogether 4,807,631 participants mentioned the relationship between birth weight and the risk of ALL; 3 including 4,143,450 participants reported the association between birth weight and the risk of AML; and 4 containing 2,261,005 participants stated the relationship between birth weight and the risk of total leukemia. More details about the baseline characteristics are displayed in Table 1. In addition, all the included studies had a score ≥6 stars and were considered as high-quality studies, as shown in Supplementary Table 1.

**Table 1:** The detailed baseline information of 28 observational studies.

**Author, year**	**Country**	**Sample size**	**Birth or diagnosed period**	**Birth weight categories (g)**	**Types of leukemia**	**Ascertainment of leukemia**	**Maximum adjusted variables**
**Cohort studies**							
Stacy et al. (37)	USA	1,877,078	2003–2015	<2,000; 2,000–2,499; 3,000–3,499; 3,500–3,999; 4,000–4,499; ≥4,500	Total	International classification of childhood cancer	Maternal age, race, sex at birth, gestational age
Paltiel et al. (38)	Israel	88,829	1964–1976	≤2,999; 3,000–3,499; 3,500–3,999; ≥4,000	Total, ALL, AML	ICD-9	Maternal origin, mother age, father age, gender, socioeconomic status
Heck et al. (39)	China	2,079,037	2004–2014	<2,500 g; 2,500–3,999 g; ≥4,000	ALL, AML	ICD-9 ICD-O-3	Mother's age, father's age, family income, urbanization level of residence at birth
Lee et al. (40)	Singapore	229,248	1992–1999	2,500–3,500; >3,500	Total, ALL	ICCC	Gender, gestational age, birth order, maternal age
Murray et al. (41)	UK	434,933	1971–1986	<3,500 g; ≥3,500	ALL	Medical records	Maternal age, birth order, Down's syndrome, gestational age, gender, social class
Spracklen et al. (42)	USA	65,850	1993–1998	<2,721; 2,721–3,624; 3,624–4,490; ≥4,490	Total	Medical records	Age, race, education, normalized socioeconomic status, BMI, smoking status, alcohol use
Westergaard et al. (43)	Denmark	1,975,584	1968–1992	<2,510; 2,510–3,009; 3,010–3,509; 3,510–4,009; 4,010–4,509; ≥4,510	ALL, AML	ICO	Gender, gestational age, maternal age, birth order calendar period
**Case-control**							
Jiménez-Hernández et al. (20)	Mexico	Case 1,455 Control 1,455	2010–2015	<2,500; 2,500–3,499; 3,500–4,000; >4,000	ALL, AML	Medical Records	Child's sex, overcrowding index, birth order, mother's age at the time of pregnancy
Barahmani et al. (24)	USA	Case 575 Control 11,379	1995–2003	≤2,500; 2,500–3,999; ≥4,000	ALL	Medical records	Infant gender, maternal age
Dorak et al. (28)	UK	Case 732 Control 3,723	1968–1992	≤2,500; 2,500–2,999; 3,000–3,499; 3,500–3,999; ≥4,000	ALL	Medical records	None
Hjalgrim et al. (29)	Denmark	Case 2,204 Control 10,745	1984–1999	<1,500; 1,500–1,999; 2,000–2,499; 2,500–2,999; 3,000–3,499; 3,500–3,999; 4,000–4,499; ≥4,500	ALL, AML	FAB classification M0–M7	None
Koifman et al. (6)	Brazil	Case 201 Control 440	1999–2005	<2,500; 2,500–2,999; 3,000–3,499; 3,500–3,999; ≥4,000	Total, ALL, AML	Medical records	Gender, income, maternal age, hormone intake, pesticide exposure during pregnancy
Groves et al. (25)	USA	Case 401 Control 1,592	1995–2002	≤2,500; 2,500–3,999; ≥4,000	ALL	Medical records	None
Ma et al. (7)	USA	Case 366 Control 460	1995–2002	≤2,500; 2,500–3,999; ≥4,000	ALL, AML	Medical records	Household income, maternal education
Oksuzyan et al. (9)	USA	Case 5,788 Control 5,788	1988–2008	<2,500; 2,500–3,000; 3,000–3,500; 3,500–4,000; 4,000–4,500; ≥4,500	Total, ALL, AML	Medical records	Gestational age, birth order, mother's age, father's education, child's race, and payment source for delivery
Ou et al. (30)	USA	Case 1,842 Control 1,986	1989–1993	≤3,000; 3,001–3,500; 3,501–4,000; >4,000	ALL	Medical records	Socioeconomic status, maternal age, race
Podvin et al. (31)	USA	Case 595 Control 5,950	1981–2002	≤2,500; 2,500–3,999; ≥4,000	Total, ALL, AML	ICD-O-3	Maternal age
Reynolds et al. (32)	USA	Case 1,728 Control 2,802	1988–1997	≤2,500; 2,500–3,999; ≥4,000	ALL, AML	Medical records	Gestational age
Smith et al. (34)	UK	Case 3,651 Control 6,337	1991–1996	≤2,500; 2,500–3,999; ≥4,000	Total, ALL, AML	Medical records	study region, sex and age
Sprehe et al. (35)	USA	Case 2,254 Control 11,734	1995–2003	≤2,500; 2,500–3,999; ≥4,000	Total, ALL, AML	Medical records	Birth year, gestational age, maternal age
Yeazel et al. (36)	USA	Case 3,711 Control 816	1982–1989	≤2,797; 2,798–3,291; 3,292–3,547; 3,548–3,859; >3,859	ALL, AML	Medical records	Maternal age, birth order, gestational age, gender
Roman et al. (26)	UK	Case 128 Control 286	1962–1992	<2,500; 2,500–3,500; >3,500	Total, ALL, AML	Medical records	Gender, age, study region
Schüz et al. (21)	Germ total	Case 755 Control 2,057	1992–1994	≤2,500; 2,500–3,999; ≥4,000	ALL, AML	Medical records	Maternal age, degree of urbanization, and socioeconomic status
McLaughlin et al. (23)	USA	Case 148 Control 9,667	1985–2001	<2,500; 2,500–2,999; 3,000–3,499; 3,500–3,999; 4,000–4,499; ≥4,500	ALL, AML	ICD-O-3	Birth year, gender, race, and ethnicity, maternal age
Jourdan-Da Silva et al. (22)	USA	Case 473 Control 567	1995–1998	<2,500; 2,500–2,999; 3,000–3,499; 3,500–3,999; ≥4,000	ALL, AML	Medical records	Gender, age at diagnosis, region of residence at diagnosis
Cnattingius et al. (27)	Sweden	Case 610 Control 3,061	1973–1989	<1,500; 1,500–1,999; 2,000–2,499; 2,500–2,999; 3,000–3,499; 3,500–3,999; 4,000–4,499; ≥4,500	ALL	ICD-7	Gestational age
Okcu et al. (8)	USA	Case 104 Control 245	1995	2,500–3,999; ≥4,000	Total, ALL	Medical records	Year of birth, gender, gestational age, maternal age, Oksuzyan tobacco use, parity, race/ethnicity
Savitz et al. (33)	USA	Case 68 Control 208	1976–1983	≤2,500; 2,500–3,999; ≥4,000	ALL	Medical records	None

*ICD-9, International Classification of Diseases, Ninth Revision codes; ICD-O-3, International Classification of Diseases for Oncology, Version 3; ICD-7, International Classification of Diseases Seventh Revision; ICO, International Classification of Diseases for Oncology. FAB classification M0–M7, French American, and British classification system; ICCC, International Childhood Cancer Classification*.

### Meta-Analysis

#### Total Leukemia

**Figure 2:**
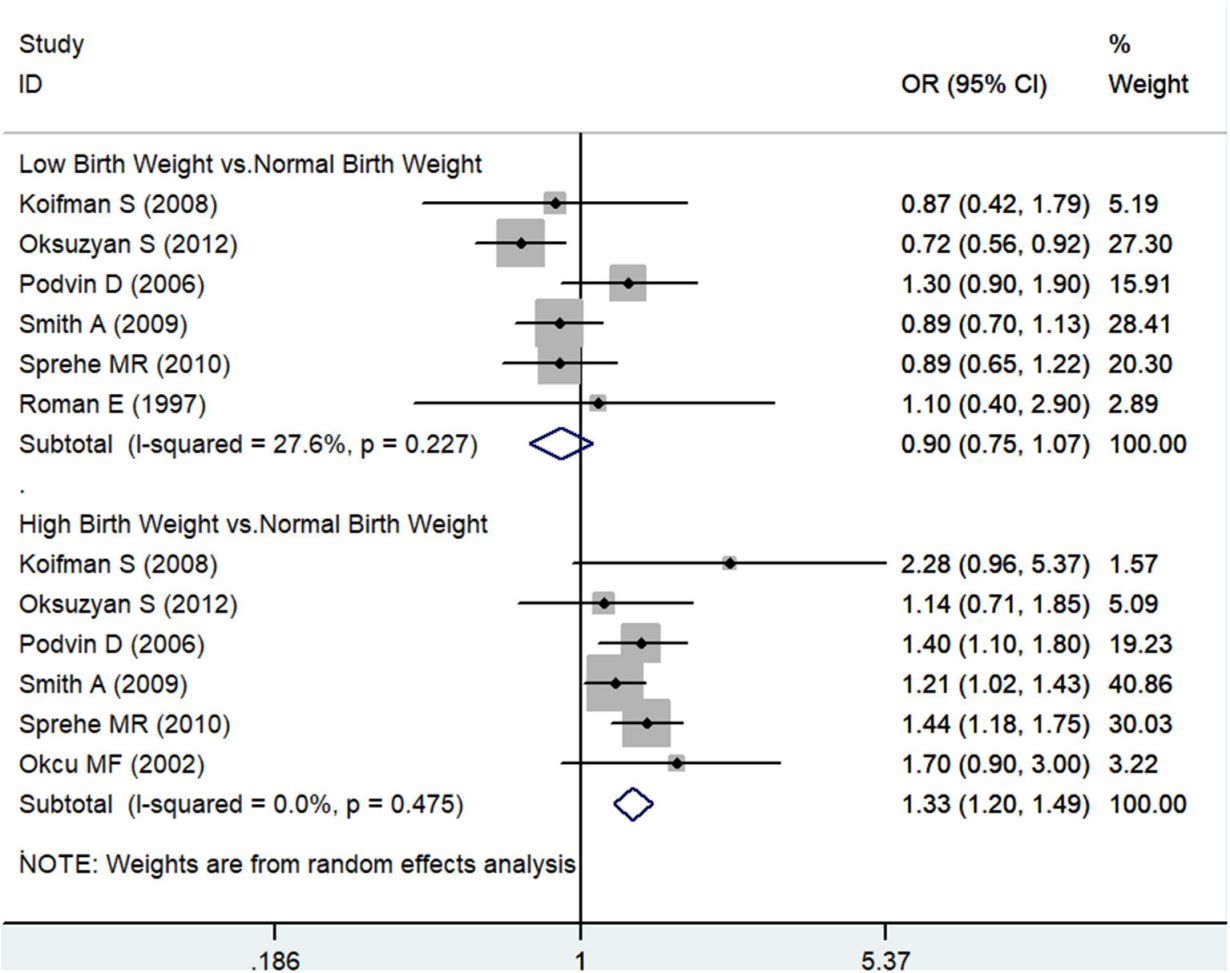
The odds ratios for low birth weight, high birth weight, and total leukemia.

As presented in Figure 2, no obvious evidence was found between LBW and the risk of total leukemia from the pooled analysis of enrolled case-control studies (OR 0.90, 95% CI 0.75–1.07; *I*^2^ 27.6%). Compared with the NBW population, the HBW population might have an increased risk of leukemia (OR 1.33, 95% CI 1.20–1.49; *I*^2^ 0%). The potential publication bias was evaluated by the funnel plot, as shown in Supplementary Figure 1A. Visually, the funnel plot was symmetrical, and both Begger's (*p* = 1.00) and Egger's (*p* = 0.881) tests did not reveal any evidence of publication bias. Moreover, sensitivity analysis was conducted by removing one study each time, and the pooled results showed little change, as shown in Supplementary Figure 2.

Compared with the NBW population, no obvious evidence was detected between LBW/HBW and the risk of leukemia from the pooled analysis of 4 cohort studies (RR 0.94, 95% CI 0.68–1.30; *I*^2^ 0% for LBW; RR 1.27, 95% CI 0.89–1.83; *I*^2^ 31% for HBW), respectively, as presented in Supplementary Figure 3A. Due to the limited number of available studies, publication bias test and sensitivity analysis were not conducted further.

According to Supplementary Figure 4A, the dose-response analysis from 4 cohort studies showed that the risk of leukemia did not increase with the increase in birth weight.

#### ALL

**Figure 3:**
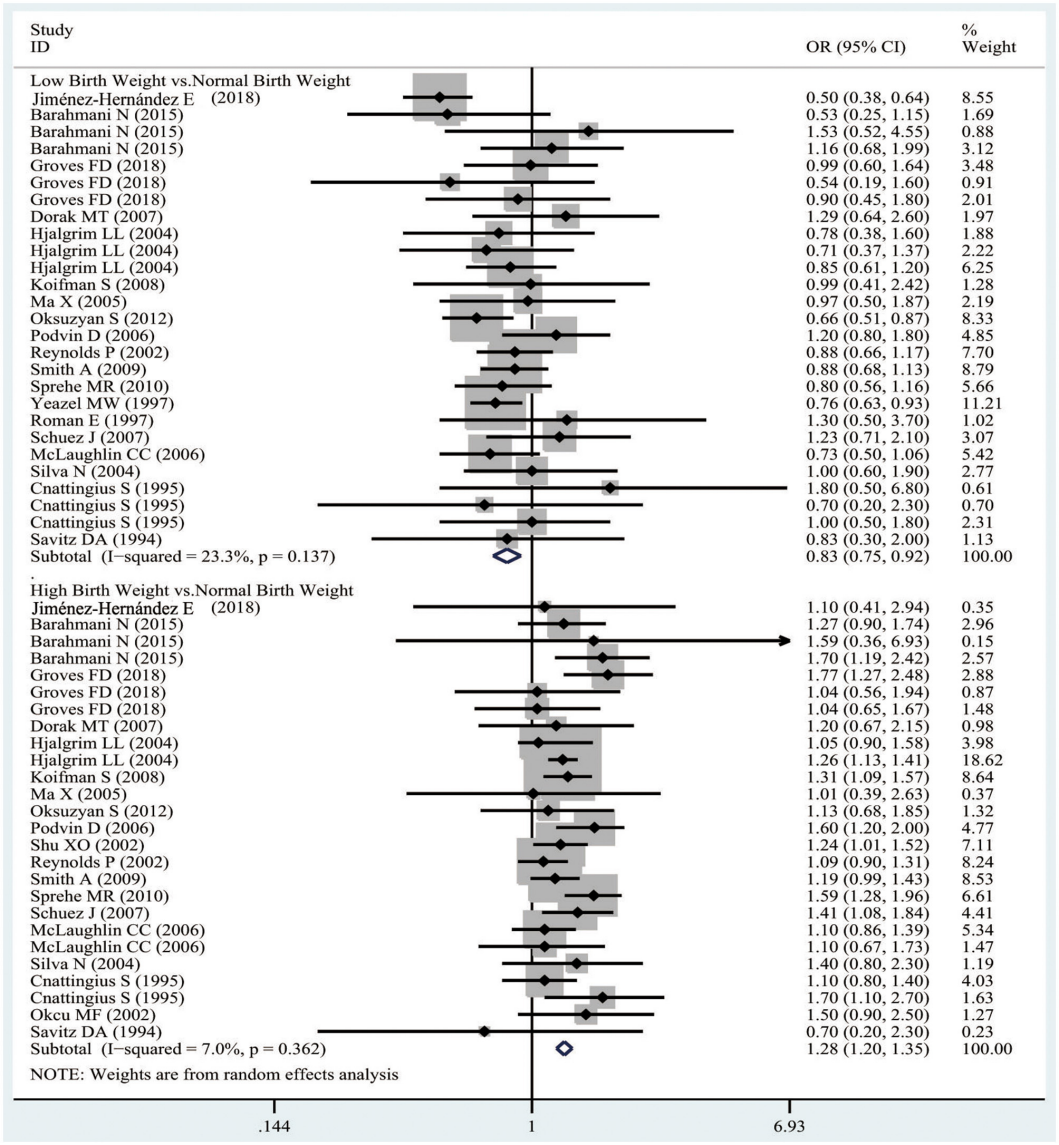
The odds ratios for low birth weight, high birth weight, and ALL.

It was demonstrated from Figure 3 that, there was obvious evidence supporting that LBW was related to a decreased risk of ALL from the pooled analysis of case-control studies (OR 0.83, 95%CI 0.75–0.92; *I*^2^ 23.3%). However, compared with the NBW population, the HBW population might have an increased risk of ALL (OR 1.28, 95%CI 1.20–1.35; *I*^2^ 7%). In addition, the publication bias was evaluated by the funnel plot, as presented in Supplementary Figure 1B. Visually, the funnel plot was asymmetrical, and both Begger's (*p* = 0.203) and Egger's (*p* = 0.256) tests revealed no obvious evidence of publication bias. Also, sensitivity analysis was conducted by removing one study each time, and the pooled results showed slight change, as shown in Supplementary Figure 5.

Similar results were obtained the pooled analysis of cohort studies, suggesting that the LBW population had a decreased risk of ALL compared with the NBW population (RR 0.66, 95% CI 0.47–0.93; *I*^2^ 0%), and the HBW population had an increased risk of ALL (RR 1.49, 95% CI 1.16–1.91; *I*^2^ 0%), as observed from Supplementary Figure 3B. Due to the limited number of available studies, the publication bias test and sensitivity analysis were not conducted further.

As observed from Supplementary Figure 4B, the dose-response analysis of 5 cohort studies showed that the risk of ALL significantly increased when the birth weight increased from 1,750 to 5,000 g.

#### AML

**Figure 4:**
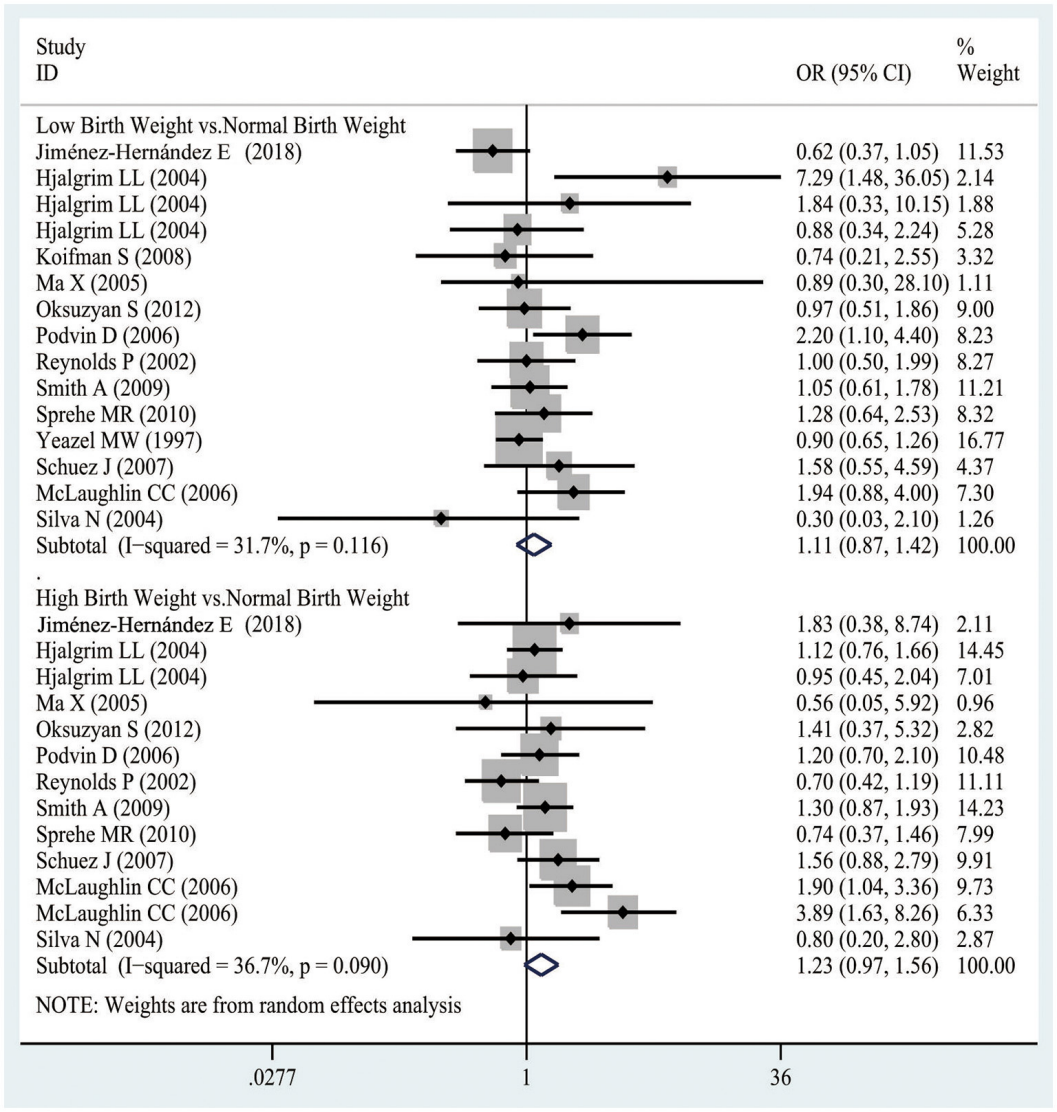
The odds ratios for low birth weight, high birth weight, and AML.

In Figure 4, there was no obvious evidence supporting the relationship between LBW and the risk of AML, as evidenced from the pooled analysis of case-control studies (OR, 1.11 95% CI 0.87–1.42; *I*^2^ 31.7%). Similarly, compared with the NBW population, the HBW population was not associated with an increased risk of AML (OR 1.23, 95% CI 0.97–1.56; *I*^2^ 36.7%). Besides, the publication bias was assessed by the funnel plot, as displayed in Supplementary Figure 1C. Visually, the funnel plot was asymmetrical, meanwhile, Begger's (*p* = 0.650) and Egger's (*p* = 0.434) tests did not indicate any evidence of publication bias. Also, sensitivity analysis was conducted by removing one study each time, and the pooled results showed little change, as shown in Supplementary Figure 6.

Results from the pooled analysis of cohort studies demonstrated that the HBW population had an increased risk of AML compared with the NBW population (RR 1.88, 95% CI 1.10–3.22; *I*^2^ 0%), as observed from Supplementary Figure 1. Due to the limited number of available studies, the publication test and sensitivity analysis were not conducted further.

However, the dose-response analysis of 3 cohort studies suggested that the risk of AML did not significantly increase with the increase in birth weight, as shown in Supplementary Figure 4C.

#### Subgroup Analysis and Meta-Regression

A moderate heterogeneity was found in the association between birth weight and AML, therefore, subgroup analysis and meta-regression were performed based on the features below, including child age, sample size, publication year, country, and study quality (Table 2). According to subgroup analysis, child age and study quality might be the potential sources of heterogeneity between LBW and AML, whereas sample size, child age, publication year, country, and study quality might be the potential sources of heterogeneity between HBW and AML. However, no potential source of heterogeneity was detected through meta-regression.

**Table 2:** Subgroup analysis for birth weight and AML.

**Items**	**Low birth weight**				**High birth weight**			
	***n***	**OR, 95% CI; *I*^**2**^**	***p* ^a^**	***p* ^b^**	***n***	**OR, 95% CI; *I*^**2**^**	***p* ^a^**	***p* ^b^**
**Sample size (cases)**				0.414				0.577
≥500	9	1.1 (0.84–1.44); 38.5%	0.093		8	1.1 (0.91–1.33); 0%	0.532	
<500	4	1.12 (0.53–2.37); 20.8%	0.285		3	1.86 (0.93–3.74); 45.9%	0.136	
**Children age**				0.537				0.632
10+ years	9	1.08 (0.79–1.49); 41.5%	0.072		8	1.22 (0.99–1.50); 0%	0.961	
≤10 years	4	1.25 (0.82–1.85); 0%	0.498		3	1.36 (0.64–2.87); 81.8%	0.001	
**Published year**				0.996				0.460
≥2010	3	0.87 (0.57–1.34); 31.7%	0.231		3	0.94 (0.53–1.66); 0%	0.469	
<2010	10	1.22 (0.91–1.64); 29.9%	0.153		8	1.28 (0.97–1.67); 45.6%	0.056	
**Country**				0.642				0.944
America	10	1.04 (0.79–1.38); 33.3%	0.141		8	1.25 (0.82–1.91); 54.4%	0.025	
Europe	3	1.40 (0.8–2.45); 33.4%	0.198		3	1.23 (0.97–1.56); 0%	0.707	
**Study quality**				0.185				0.272
8–9	8	1.41 (0.97–2.05); 23.8%	0.224		7	1.38 (1.08–1.77); 28.1%	0.195	
6–7	5	0.89 (0.71–1.12); 0%	0.541		4	0.8 (0.54–1.17); 0%	0.559	

*P^a^ for heterogeneity within each subgroup. P^b^ for heterogeneity between subgroups with meta-regression analysis*.

## Discussion

This meta-analysis suggested that LBW might not increase the risk of total leukemia, ALL, or AML compared with NBW. In contrast, the risk of ALL significantly decreased in the LBW population. In the HBW population, HBW might increase the risk of total leukemia and AML, but there was no dose-response relationship. In addition, compared with the NBW population, the HBW population had an increased risk of ALL, and similar results were obtained from the dose-response analysis of birth weight from 1,750 to 5,000 g.

Birth weight is determined by the nutritional, metabolic, and endocrine differences in the intrauterine environment, and is necessarily closely related to maternal prenatal health and nutritional status. The study by Wiemels et al. showed that chromosomal translocation in acute leukemia, a genetic event, might begin *in utero* (44). In addition, birth weight has been reported to be associated with multiple growth factors in the intrauterine environments, like insulin-like growth factor-1 (IGF-1), sex steroid hormones, and insulin-like growth factor II (IGF-1I) (45, 46). *In utero*, growth factors are related to the increased total number of stem cells, which may increase the risks of transformation into tumor cells and leukemia (47). Moreover, IGF-1 is an important embryonic growth factor that increases the stem cell pools in humans and animals. Further, several other hormones and growth factors related to birth weight and stem cell size, including IGF binding protein-3, estriol, and testosterone, can significantly increase the stem cell pool (45).

Birth weight and the risk of subsequent leukemia are often the consequences of multiple reproductive factors including gestational age, race, diet, and micronutrients. As reported in the study by Barahmani et al., the offspring of Hispanic whites with large gestational age were associated with a 50% increased risk of ALL compared to the non-Hispanic whites (24). Besides, a study from Denver indicates that animal foods are rich in iron, while plant foods may hinder iron absorption, and the increased iron absorption may increase the risk of ALL in children (48). Meanwhile, another meta-analysis shows that an increased maternal iron intake by 10 mg per day is associated with an increased offspring birth weight by 15.1 g (49).

Although some existing biological evidence suggests that a larger birth size may indicate that more cells are at risk of carcinogenesis (50, 51), the exact mechanism remains unclear. LBW may reflect a poor intrauterine environment or impaired fetal nutrition. In addition, it may also be associated with certain complications during the maternal pregnancy, such as intrauterine growth restriction (IUGR), preeclampsia, or preterm delivery. However, there is still no well-understood mechanism to explain this negative association of LBW with ALL.

Noteworthily, the present study has the following strengths. First, this was the first dose-response analysis that quantitatively assessed the association between birth weight and the risk of leukemia. Meanwhile, all the available studies were included for qualitative analysis, which verified and supplemented the dose-response analysis. Second, there was low inter-heterogeneity between the studies, which enabled the homogeneity between studies. Third, all the extracted ORs or RRs were adjusted by maximum variables, and the random-effect model was adopted to improve the stability of the pooled results.

Inevitably, certain limitations should also be noted in this study. First, most of the included studies were originated from America, Europe, and Asia, while there were relatively few studies from other regions, and the impact on research remained unclear. Meanwhile, the number of cohort studies was limited, which made it impossible for further analysis. Second, this meta-analysis suggested that HBW was associated with a high risk of leukemia, but whether there is a causal relationship between the two remains to be further investigated. Besides, the impacts of other potential perinatal variables (including full-term or preterm birth, birth order, breastfeeding, or feeding) and maternal characteristics (like the presence of maternal diseases and complications such as diabetes, eclampsia, maternal age, and weight) on our results remained to be further confirmed. Although all of the extracted ORs or RRs were adjusted by the maximum variables, the effects of residual confounding variables were still unknown. Third, the dose-response analyses on birth weight were conducted within the range of 1,750–5,000 g, and the results beyond this range were still unknown. Last but not least, all the populations included in the study were children and young adults (<29 years), and the effect of birth weight on the risk of leukemia beyond this range is still unknown.

## Conclusions

Overall, in children and young adults, HBW may be related to an increased risk of subsequent leukemia and AML in HBW population compared with the NBW population, but support from dose-dependent evidence is lacking. In addition, stronger evidence supports a significantly increased risk of subsequent ALL in the HBW population compared with the NBW population, whereas a decreased risk in the LBW population, and this association is also found in the dose-response. More prospective studies with large samples are still warranted in the future to validate and complement these findings.

## Data Availability Statement

The original contributions presented in the study are included in the article/Supplementary Materials, further inquiries can be directed to the corresponding author/s.

## Author Contributions

HC participated in the data collection, data review, relevant data extraction, data analysis, statistical analysis, and the writing of the manuscript. DL, QS, and YL participated in checking data extraction as well as in the data analysis, statistical analysis, and the writing of the manuscript. LW participated in checking data extraction as well as in the statistical analysis and the writing of the manuscript. All authors saw and approved the final version.

## Conflict of Interest

The authors declare that the research was conducted in the absence of any commercial or financial relationships that could be construed as a potential conflict of interest.

## Publisher's Note

All claims expressed in this article are solely those of the authors and do not necessarily represent those of their affiliated organizations, or those of the publisher, the editors and the reviewers. Any product that may be evaluated in this article, or claim that may be made by its manufacturer, is not guaranteed or endorsed by the publisher.

## Supplementary Material

Supplementary Figure 1 The plots of the funnel for birth weight and Total leukemia **(A)**, ALL **(B)**, and AML **(C)**.

Supplementary Figure 2 The sensitivity analyses for low birth weight **(A)**, high birth weight **(B)**, and Total leukemia.

Supplementary Figure 3 The RRs of birth weight and total leukemia **(A)**, AML **(B)**, and ALL **(C)**.

Supplementary Figure 4 The dose-response of birth weight and total leukemia **(A)**, AML **(B)**, and ALL **(C)**.

Supplementary Figure 5 The sensitivity analyses for low birth weight **(A)**, high birth weight **(B)**, and ALL.

Supplementary Figure 6 The sensitivity analyses for low birth weight **(A)**, high birth weight **(B)**, and AML.

Supplementary Table 1 Quality assessment of the 28 observational studies.
