# Supplementary figures and images for: Birth Weight and Subsequent Risk of Total Leukemia and Acute Leukemia: A Systematic Review and Meta-Analysis
Source: Front Pediatr. 2021 Sep 23;9:722471. doi: 10.3389/fped.2021.722471 (PMC8495325; doi:10.3389/fped.2021.722471)

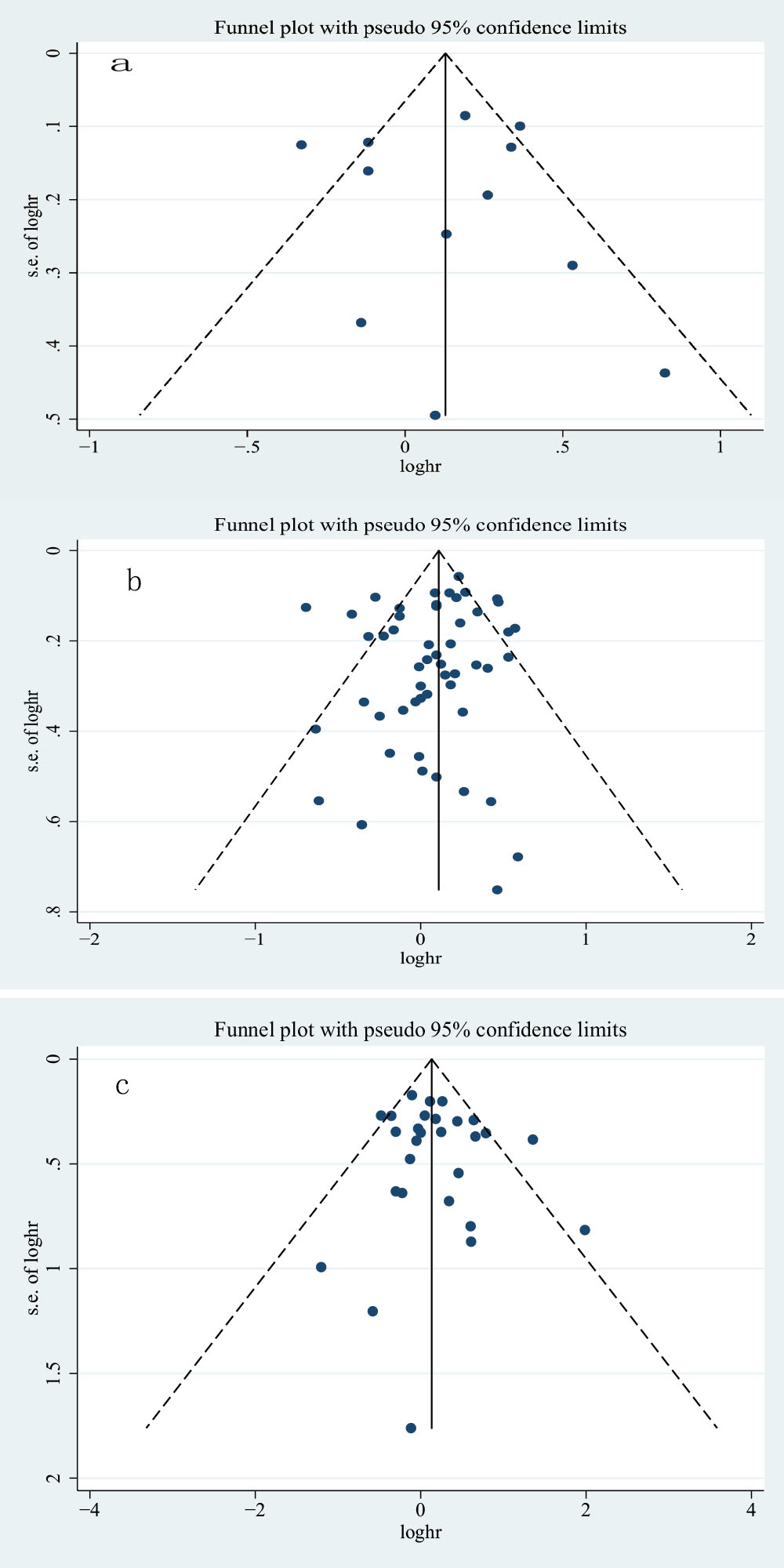

Supplement: Supplementary Figure 1 — The plots of the funnel for birth weight and Total leukemia (A), ALL (B), and AML (C). [file Image_1.JPEG]

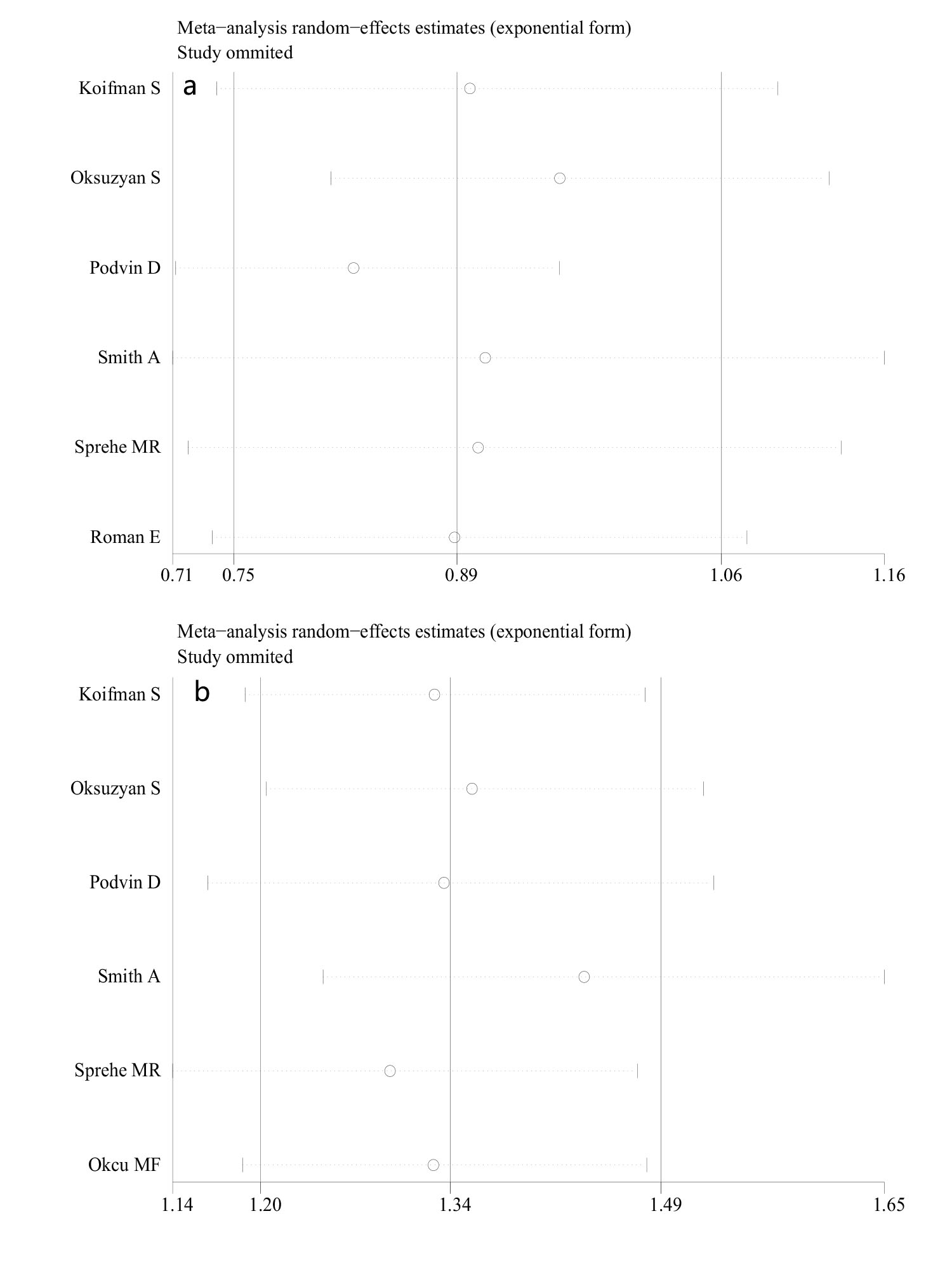

Supplement: Supplementary Figure 2 — The sensitivity analyses for low birth weight (A), high birth weight (B), and Total leukemia. [file Image_2.JPEG]

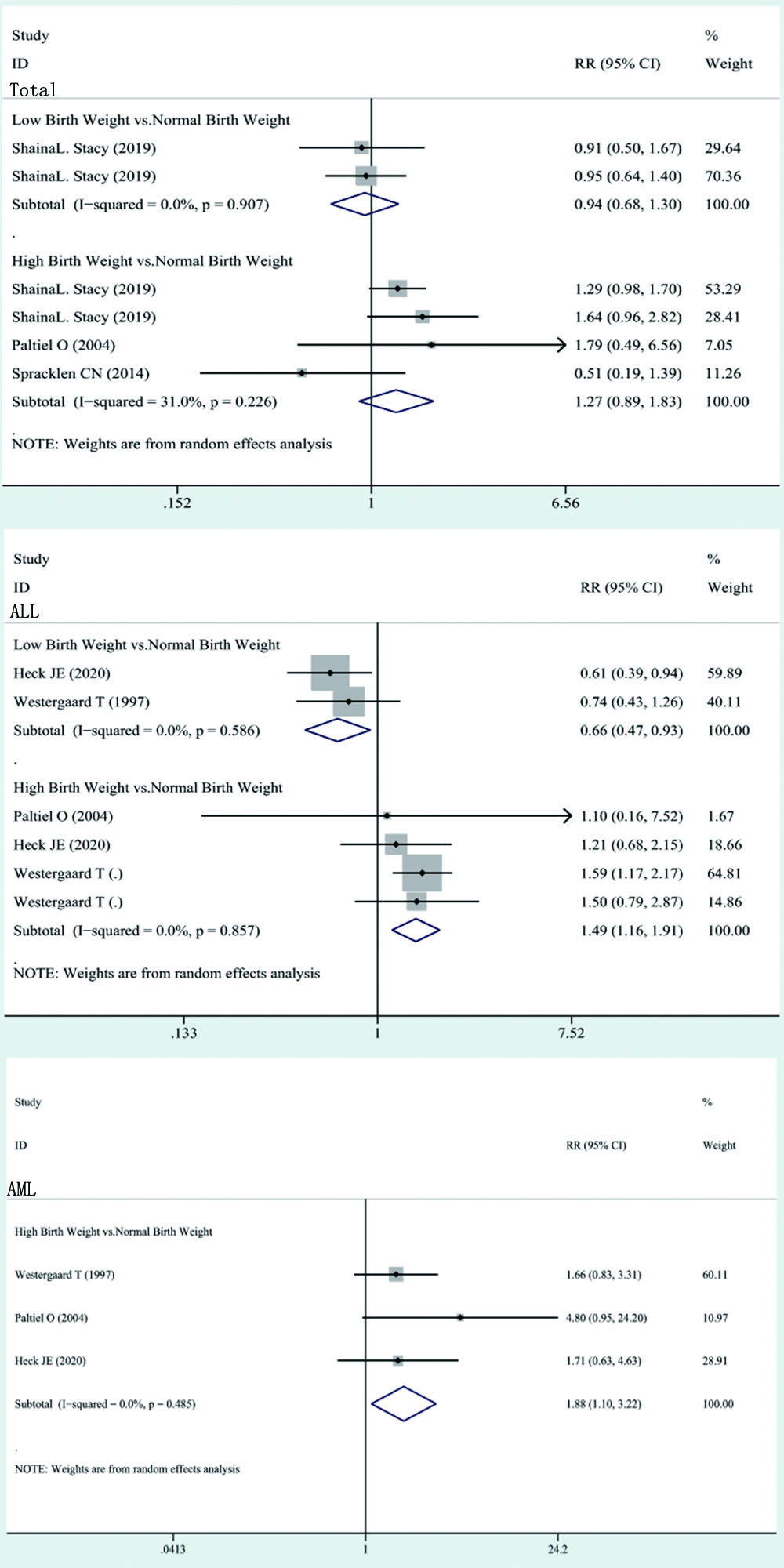

Supplement: Supplementary Figure 3 — The RRs of birth weight and total leukemia (A), AML (B), and ALL (C). [file Image_3.JPEG]

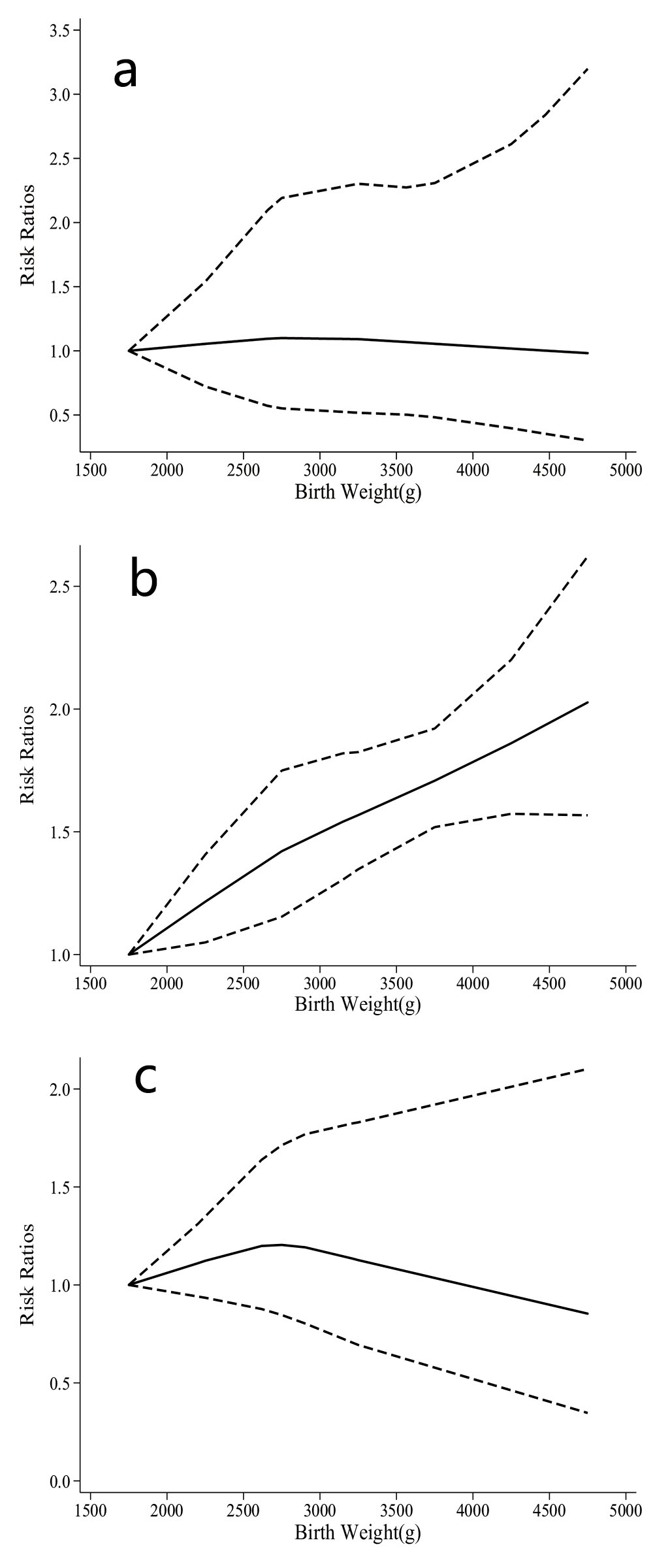

Supplement: Supplementary Figure 4 — The dose-response of birth weight and total leukemia (A), AML (B), and ALL (C). [file Image_4.JPEG]

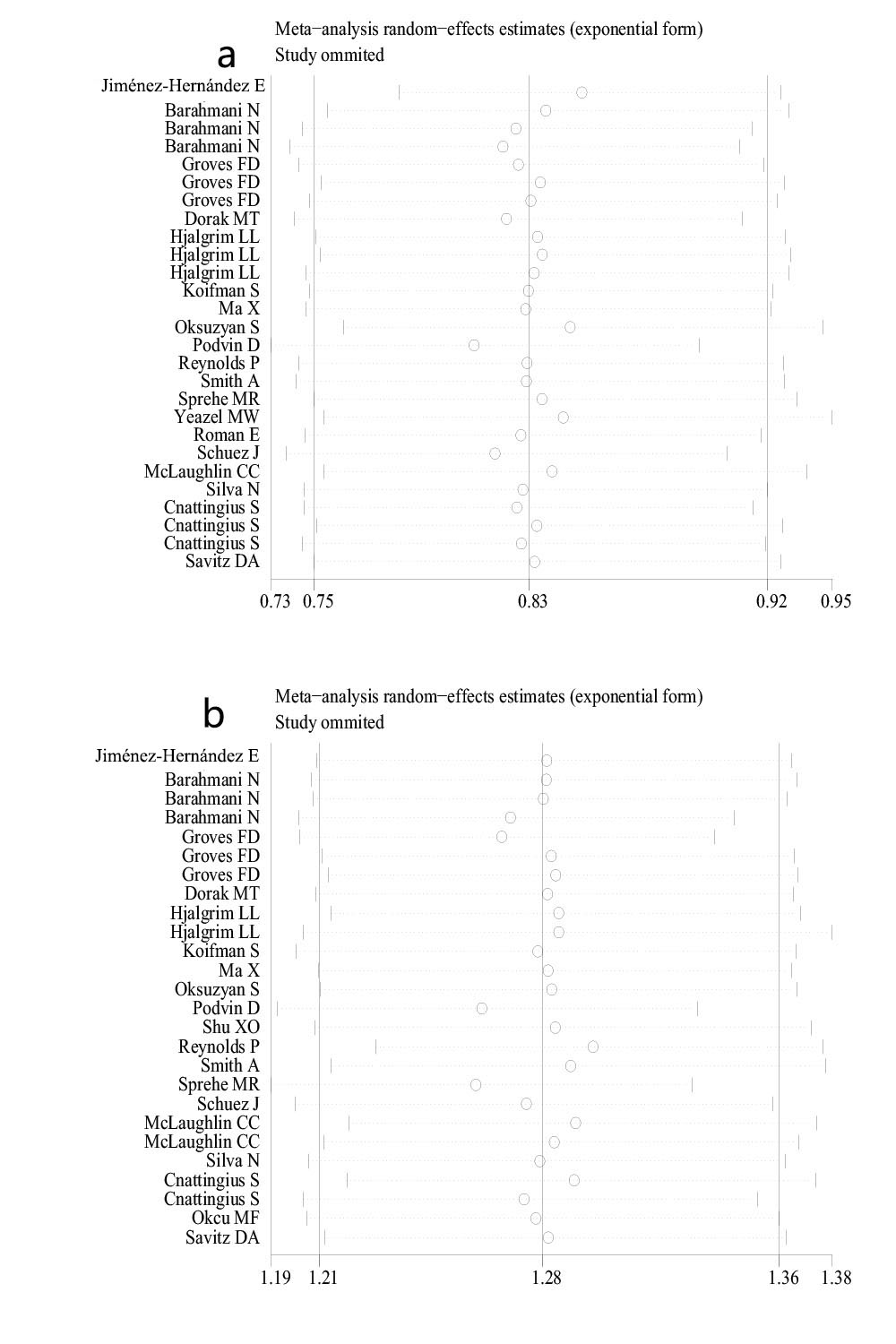

Supplement: Supplementary Figure 5 — The sensitivity analyses for low birth weight (A), high birth weight (B), and ALL. [file Image_5.jpg]

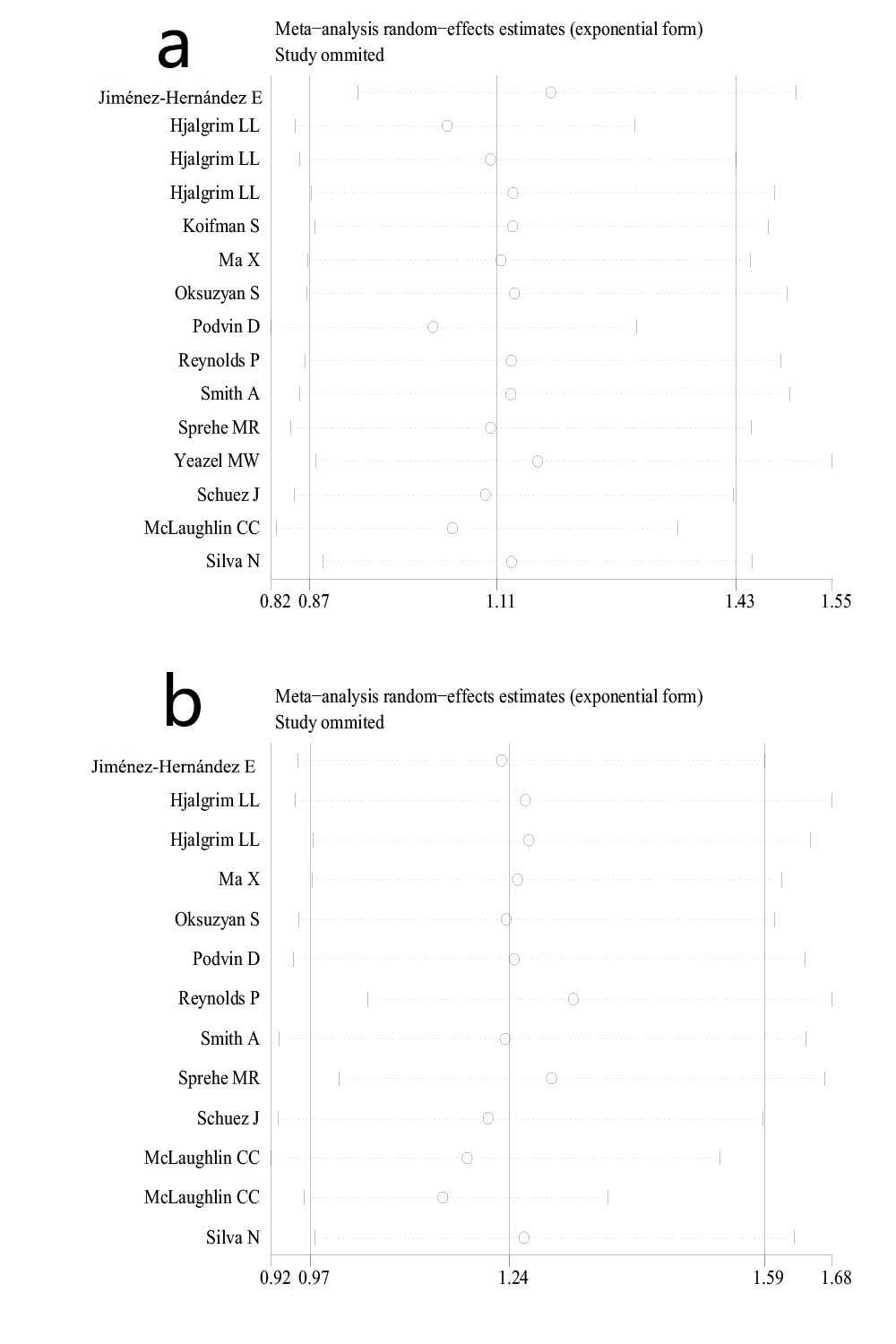

Supplement: Supplementary Figure 6 — The sensitivity analyses for low birth weight (A), high birth weight (B), and AML. [file Image_6.jpg]
