# Supplementary material for: Birth Weight and Subsequent Risk of Total Leukemia and Acute Leukemia: A Systematic Review and Meta-Analysis
Source: Front Pediatr. 2021 Sep 23;9:722471. doi: 10.3389/fped.2021.722471 (PMC8495325; doi:10.3389/fped.2021.722471)
Supplement: Supplementary Table 1 — Quality assessment of the 28 observational studies. [file Table_1.DOCX]

| ***Supplementary Table 1. Quality Assessment of the 28 observational Studies*** | | | | |
| --- | --- | --- | --- | --- |
| **Study**  **(First Author, Year)** | **Selection** | **Comparability** | **Outcome/ exposure** | **Total** |
| Stacy S,2019 | **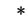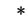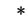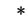** | **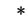** | **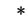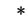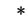** | **8** |
| Paltiel O,2004 | **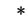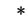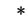** | **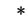** | **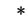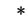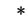** | **7** |
| Heck JE,2020 | **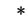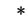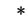** | **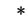** | **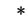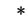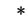** | **7** |
| Lee J,2004 | **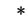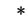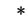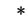** | **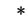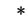** | **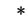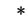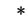** | **8** |
| Murray L,2002 | **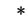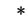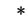** | **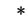** | **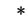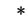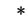** | **7** |
| Spracklen CN,2014 | **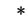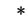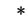** | **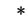** | **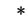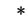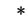** | **7** |
| Westergaard T,1997 | **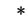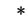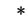** | **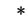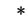** | **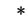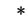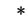** | **8** |
| Jiménez-Hernández E,2018 | **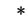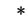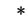** | **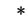** | **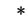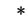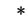** | **7** |
| Barahmani N,2015 | **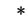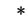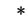** | **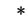** | **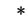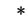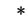** | **7** |
| Dorak MT,2007 | **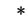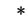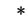** |  | **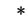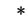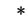** | **6** |
| Hjalgrim LL,2004 | **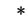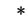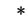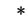** | **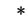** | **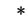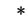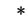** | **8** |
| Koifman S,2008 | **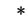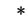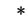** | **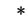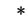** | **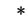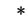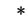** | **8** |
| Groves FD,2018 | **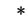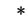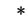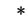** | **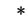** | **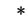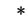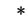** | **8** |
| Ma X,2005 | **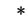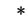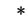** |  |  | **8** |
| Oksuzyan S,2012 |  |  |  | **7** |
| Shu XO,2002 |  |  |  | **7** |
| Podvin D,2006 |  |  |  | **8** |
| Reynolds P,2002 |  |  |  | **7** |
| Smith A,2009 |  |  |  | **8** |
| Sprehe MR,2010 |  |  |  | **7** |
| Yeazel MW,1997 |  |  |  | **7** |
| Roman E,1997 |  |  |  | **7** |
| Schuez J,2007 |  |  |  | **8** |
| McLaughlin CC,2006 |  |  |  | **8** |
| Silva N,2004 |  |  |  | **8** |
| Cnattingius S,1995 |  |  |  | **7** |
| Okcu MF,2002 |  |  |  | **8** |
| Savitz DA,1994 |  |  |  | **7** |
