# Supplementary material for: Birth Weight and Subsequent Risk of Total Leukemia and Acute Leukemia: A Systematic Review and Meta-Analysis
Source: Front Pediatr. 2021 Sep 23;9:722471. doi: 10.3389/fped.2021.722471 (PMC8495325; doi:10.3389/fped.2021.722471)
Supplement: Supplementary file 8 [file Data_Sheet_1.pdf]

Appendix 1

Pubmed

1. Search: ((("Birth Weight"[Mesh]) OR (Birth Weights[Title/Abstract])) OR (Weight, Birth[Title/Abstract])) OR (Weights, Birth[Title/Abstract]) Sort by: Publication Date
2. Search:("Neoplasms"[Mesh]) OR (((((((((((((((Neoplasia[Title/Abstract]) OR (Neoplasias[Title/Abstract])) OR (Neoplasm[Title/Abstract])) OR (Tumors[Title/Abstract])) OR (Tumor[Title/Abstract])) OR (Cancer[Title/Abstract])) OR (Cancers[Title/Abstract])) OR (Malignancy[Title/Abstract])) OR (Malignancies[Title/Abstract])) OR (Malignant Neoplasms[Title/Abstract])) OR (Malignant Neoplasm[Title/Abstract])) OR (Neoplasm, Malignant[Title/Abstract])) OR (Neoplasms, Malignant[Title/Abstract])) OR (Benign Neoplasms[Title/Abstract])) OR (Neoplasms, Benign[Title/Abstract])) OR (Benign Neoplasm[Title/Abstract])) OR (Neoplasm, Benign[Title/Abstract])) Sort by: Publication Date
3. 1 and 2 1012

Embase

1. 'birth weight'/exp
2. birthweight:ab,ti OR 'neonatal weight':ab,ti OR 'newborn weight':ab,ti OR 'weight, birth':ab,ti
3. #1 OR #2
4. 'malignant neoplasm'/exp
5. cancer:ab,ti OR 'malignant neoplastic disease':ab,ti OR 'malignant tumor':ab,ti OR 'malignant tumour':ab,ti OR 'neoplasia, malignant':ab,ti OR 'tumor, malignant':ab,ti OR 'tumour, malignant':ab,ti OR 'malignant neoplasia':ab,ti OR cancers:ab,ti
6. #4 OR #5
7. #3 AND #6 2911

Cochrane

1. MeSH descriptor: [Birth Weight] explode all trees
2. (Birth Weights):ti,ab,kw
3. 1 and 2
4. MeSH descriptor: [Neoplasms] explode all trees
5. (Neoplasm, Malignant):ti,ab,kw OR (Malignancies):ti,ab,kw OR (Cancer):ti,ab,kw OR (Malignancy):ti,ab,kw OR (Malignant Neoplasms):ti,ab,kw
6. (Malignant Neoplasm):ti,ab,kw OR (Cancers):ti,ab,kw OR (Neoplasms, Malignant):ti,ab,kw OR (Neoplasias; Tumors):ti,ab,kw OR (Tumor; Neoplasia):ti,ab,kw
7. (Neoplasm):ti,ab,kw OR (Benign Neoplasms):ti,ab,kw OR (Neoplasms, Benign):ti,ab,kw OR (Benign Neoplasm):ti,ab,kw OR (Neoplasm, Benign):ti,ab,kw
8. #4 or #5 or #6 or #7
9. #3 and #8 101
